# Sustained ocean changes contributed to sudden Antarctic sea ice retreat in late 2016

**DOI:** 10.1038/s41467-018-07865-9

**Published:** 2019-01-02

**Authors:** Gerald A. Meehl, Julie M. Arblaster, Christine T. Y. Chung, Marika M. Holland, Alice DuVivier, LuAnne Thompson, Dongxia Yang, Cecilia M. Bitz

**Affiliations:** 1 National Center for Atmospheric Research, Boulder, CO 80307 USA; 2 ARC Centre of Excellence for Climate Extremes, Monash University, Melbourne, Australia; 3 Bureau of Meteorology, Melbourne, Australia; 4 University of Washington, Seattle, WA 98195 USA

## Abstract

After nearly three decades of observed increasing trends of Antarctic sea ice extent, in September-October-November 2016, there was a dramatic decrease. Here we document factors that contributed to that decrease. An atmosphere-only model with a specified positive convective heating anomaly in the eastern Indian/western Pacific Ocean, representing the record positive precipitation anomalies there in September-October-November 2016, produces an anomalous atmospheric Rossby wave response with mid- and high latitude surface wind anomalies that contribute to the decrease of Antarctic sea ice extent. The sustained decreases of Antarctic sea ice extent after late 2016 are associated with a warmer upper Southern Ocean. This is the culmination of a negative decadal trend of wind stress curl with positive Southern Annular Mode and negative Interdecadal Pacific Oscillation, Ekman suction that results in warmer water being moved upward in the column closer to the surface, a transition to positive Interdecadal Pacific Oscillation around 2014–2016, and negative Southern Annular Mode in late 2016.

## Abstract (web summary)

In late 2016, there was a sudden and subsequently sustained decrease of Antarctic sea ice extent. Analyses of observations and a model simulation trace the causes to teleconnections from the tropics on the interannual timescale combined with decadal-timescale warming in the upper Southern Ocean.

## Introduction

**Fig. 1:**
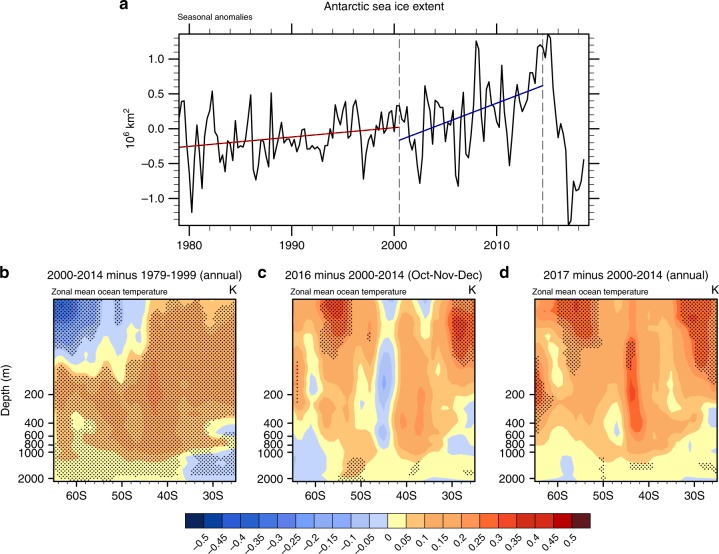
Antarctic sea ice extent and zonal mean ocean temperatures. **a** Observed seasonal anomalies of sea ice extent from 1979 through JJA 2018 (10^6^ km^2^), seasonal anomalies from the full available period 1979–August 2018; vertical dashed lines denote DJF 2000 at the start of the negative IPO period, and DJF 2014 marking the end of the negative IPO period as analyzed in ref. ^1^; trend lines are calculated through the seasonal anomalies. **b** Zonal mean temperatures (°C) from EN4 reanalysis data, base state differences for the negative IPO period (2000–2014) minus positive IPO period (1979–1999), stippling denotes significance at the 5% level; **c** as in (**b**) except for October–November–December (OND) 2016 minus 2000–2014 OND mean; **d** as in (**b**) except for 2017 annual mean minus 2000–2014 mean

The accelerated rate of Antarctic sea ice increase from 2000 to 2014 (linear trend of +0.57 +/−0.33 × 10^6^ km^2^ decade^−1^, uncertainty is represented by 5–95% confidence intervals) was nearly a factor of 5 larger than the increase from 1979 to 1999 (linear trend of +0.12 +/−0.11 × 10^6^ km^2^ decade^−1^) (ref.^1^, Fig. 1a). Previous studies^2–5^ have addressed the subsequent sudden decrease of Antarctic sea ice extent in SON 2016 (Fig. 1a) and have attributed it mainly to atmospheric processes.

The post-2000 period was when the interdecadal Pacific oscillation (IPO) was in its negative phase with below-normal SST anomalies in the tropical Pacific on decadal timescales associated with a slowdown in the rate of global warming^6,7^. The previous 1979–2000 period saw the IPO in its positive phase with an acceleration of global warming^7^. A number of studies have attempted to attribute factors that could have produced the expansion of Antarctic sea ice in the post-1979 period when the multi-model ensemble average from the CMIP set of models showed a decrease^1^. Evidence from observations and model sensitivity experiments has supported the idea that the expansion of Antarctic sea ice extent was associated with atmospheric teleconnection patterns driven from various areas in the tropics and associated surface wind anomalies at high southern latitudes^8–13^, as well as a freshening of the Southern Ocean^14^. Subsequent evidence showed that these teleconnection patterns, which affected the acceleration of the increase in Antarctic sea ice extent after 2000, were driven predominantly by negative convective heating anomalies in the tropical Pacific. These were associated with the negative phase of the IPO, with secondary contributions from convective heating anomalies in the tropical Atlantic and SPCZ regions^1^.

**Fig. 2:**
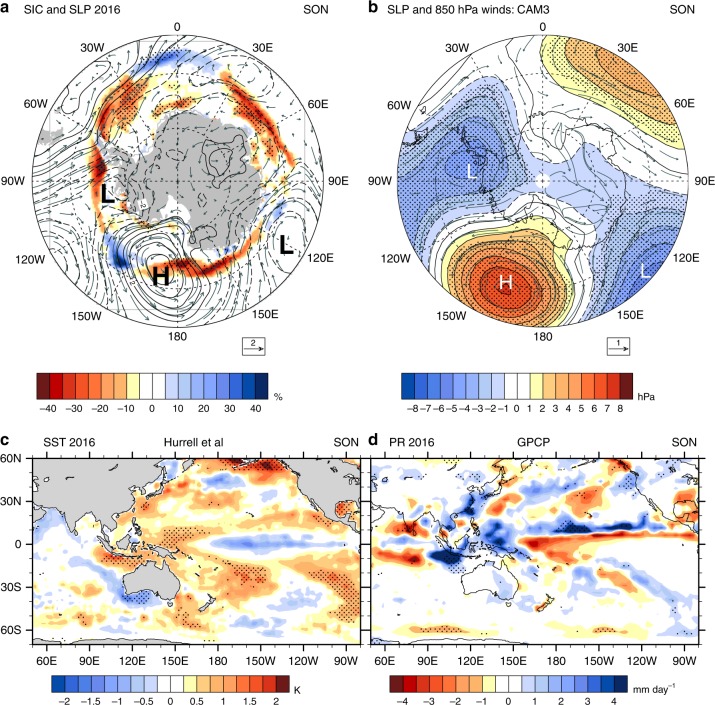
Teleconnections from tropics to southern mid- and high latitudes. **a** SON 2016 sea ice concentration anomalies (colors, %, compared to 1981–2010 base period^31^, stippling indicates 5% significance level), SLP (hPa, contour interval 1 hPa, zero line excluded; values greater in magnitude than about 2 hPa are significant at the 5% level^32^), and 850 hPa wind anomaly vectors (2 m sec^−2^ scaling arrow at the lower right) from ERA-Interim compared to 1981–2010 base period; observed SAM index for SON 2016 of −0.52; **b** atmospheric model experiment with positive specified convective heating anomaly centered at Eq, 120°E, SLP (hPa, colors and contour interval 1 hPa, stippling denotes significance at the 5% level; 1 m sec^−2^ scaling arrow for 850 hPa wind anomaly vectors at the lower right, note the difference to scaling in (**a**)) for 30-year SON average minus 100-year SON control run average (see Methods); **c** SST anomalies (°C)^26^ for SON 2016 compared to 1981–2010 base period; **d** precipitation anomalies for SON 2016 compared to 1981–2010 base period from GPCP, mm day^−1^

Then, in late 2016, there was a sudden decrease of Antarctic sea ice. Possible factors contributing to this decrease include a persistent zonal wave number 3 pattern from early winter through spring indicating enhanced meridional flow^4^, and negative values of the SAM index toward the end of the year, with a near-record (since 1957) negative SAM anomaly in November^2,3^. This was associated with record (since 1979) zonal mean easterly wind anomalies in Nov–Dec 2016 near 60–65°S of about −4 m sec^−1 ^^5^ that produced positive wind stress curl anomalies. Additionally, the Indian Ocean Dipole for Sept–Oct 2016 was at a record negative dipole mode index (DMI) value (since 1979) of about −1.0^5^. The positive SST anomalies in the tropical eastern Indian Ocean and far-western Pacific associated with that large negative DMI value produced record (for the 2000s) enhanced convection during SON. This is indicated by a record low outgoing longwave radiation (OLR) anomaly for the area 90°E–150°E, 15°S–15°N of nearly −13 Wm^−2^ (Supplementary Figure 1a) and an associated record precipitation anomaly (for the 2000s) averaged over that region of about +1.4 mm day^−1^ (Supplementary Figure 1b) with maximum values approaching +5 mm day^−1^ (Fig. 2d).

The IPO in its negative phase can produce anomalous negative SLP anomalies south of about 60°S and positive SLP anomalies north of that latitude on decadal timescales, with stronger westerlies near 60°S and below normal SSTs there (ref.^1^, and Supplementary Figure 2). Therefore, if the IPO transitioned to positive in the 2014–2016 time frame (as has been indicated by initialized predictions^15,16^ as well as subsequent analyses of observations^17,18^), decadal climate variability also could have contributed to the changes in upper ocean temperatures around Antarctica that played a role in the sudden decrease in Antarctic sea ice extent^3^. Here we analyze the observations and a model sensitivity experiment to show that a combination of factors contributed to the sudden and sustained decrease in Antarctic sea ice extent that started in late 2016. First, teleconnections from strong tropical convection in the eastern Indian Ocean produced surface wind anomalies in the vicinity of the Antarctic sea ice that contributed to its decreased extent. Second, decadal-timescale trends of strengthened circum-Antarctic westerlies associated with the negative phase of the IPO and positive phase of the SAM moved warm subsurface water upward in the water column due to Ekman suction. Third, a negative phase of the SAM in SON 2016 in the context of a transition to a positive phase of the IPO produced warm SSTs that completed a warming of the upper 600 m over much of the circum-polar ocean around Antarctica to sustain the decreases of Antarctic sea ice extent after late 2016.

## Results

### Tropical teleconnections in 2016

In the first part of 2016 (DJF 2016, MAM 2016, Supplementary Figure 3a,b), zonal mean SSTs south of 50°S were about 0.5 °C colder than the average temperature of 2000–2014 (Fig. 1b). However, in JJA 2016, zonal mean SST anomalies near 60–65°S were positive (Supplementary Figure 3c) and there were indications that Antarctic sea ice had already started to retreat^4^. Then, by SON 2016, zonal mean SSTs were anomalously positive by nearly +0.5 °C compared to the average of 2000–2014 south of 50°S (Supplementary Figure 3d). This was not a one-season anomaly, since sea ice extent remained well below values averaged over the 2000–2014 period after SON 2016 (Fig. 1a). Zonal mean annual mean ocean temperature anomalies for 2017 remained positive in the upper ocean south of about 45°S, and significantly positive at the 5% level from about 50 to 60°S above roughly 150 m, and from about 62 to 65°S at depths of 150–300 m (Fig. 1d).

There are indications from observations that an anomalous Rossby wave response in the atmosphere, emanating from the tropical eastern Indian Ocean/western Pacific where there were positive SST anomalies in that region (Fig. 2a, c), produced surface wind anomalies around Antarctica that could have contributed to the retreat of sea ice in southern spring 2016^5^. The observed SLP, surface wind, and sea ice concentration anomalies for SON 2016 (Fig. 2a) show sea ice retreat occurring roughly where there are anomalous northerly component surface winds acting to drive ice toward the south with associated warm air advection^19^. This occurs mainly on the eastern flank of an elongated anomalous low stretching from the Amundsen Sea toward the Weddell Sea, on the western flank of an anomalous high in the Ross Sea, and on the eastern side of an anomalous low in the southern Indian Ocean. To trace this anomalous SLP and surface wind anomaly pattern back to the tropics, we center a positive convective heating anomaly at the equator, 120°E (see Methods) in an atmosphere model. This convective heating anomaly is at the location where the positive SST anomalies of nearly +2 °C near Sumatra and Java occurred (Fig. 2c) along with record OLR and precipitation anomalies (Fig. 2d, Supplementary Figure 3). The resulting anomalous atmospheric circulation pattern (Fig. 2b) shows a strong correspondence to the observations, with an anomalous elongated low in the Amundsen Sea extending to the Weddell Sea, an anomalous high in the Ross Sea sector, and an anomalous low south of Australia. The magnitude of the forcing from the tropics (precipitation anomalies up to +5 mm day^−1^) and the response in the mid-latitudes (SLP anomalies of up to about +/−5 hPa) are comparable in both the observations and the model simulation (Fig. 2). The observed SAM index averaged for SON 2016 is −0.52 (standard deviation of SON values of the SAM index from 1979 to 2016 is 1.89), and the SAM index for the SON model simulation is −1.12. The preponderance of negative v-component surface winds is similar in the model simulation compared to observations (Supplementary Figure 4). Therefore, for the SON 2016 season, evidence from a model simulation indicates that there is a strong connection between the anomalous convective heating in the tropical eastern Indian/western Pacific and the teleconnection pattern of surface winds around Antarctica seen in observations^5^ that contributed to the sea ice retreat.

In looking at a subsequent cold season, JJA 2017, the SSTs remained warm in the far western tropical Pacific (Supplementary Figure 5a) with continued positive precipitation anomalies there (Supplementary Figure 5c,d). The results from the atmospheric model experiment described above but for JJA show a circum-global teleconnection pattern (Supplementary Figure 5b) with comparable magnitude to the observations (Supplementary Figure 5a) with anomalous lows near the Amundsen Sea and south of Australia, and anomalous highs in the Ross Sea, southern Indian Ocean, and South Atlantic. There is also a strong association between anomalous northerly component surface winds and regions of sea ice retreat as there was in SON 2016 (Fig. 2a). The preponderance of anomalous negative model v-component surface zonal winds also is comparable to the observations (Supplementary Figure 6).

It has been suggested that the strongly negative SAM during spring and summer 2016 contributed to sustained sea ice reductions through autumn, 2017, via warming of Southern Ocean SSTs^20^. However, that study found no relationship between summertime SAM and wintertime sea ice extent, and predicted that the size of the sea ice extent anomaly in 2017 would reduce as the sea ice marched toward its maximum extent in September. Yet as Fig. 1a depicts, while the sea ice has recovered somewhat from the record low values in 2016, it has remained well below the 2000s average for the subsequent seven seasons. The most recent full season for which there are data available (JJA 2018) is still showing a negative anomaly of −0.45 × 10^6^ km^2^. In the 2000s, the three previous years of the lowest sea ice extent (2002, 2006, and 2011) all showed recovery in a few seasons. In 2002, the negative values of −0.15 × 10^6^ km^2^ in DJF and −0.52 × 10^6^ km^2^ in MAM dropped to −0.78 × 10^6^ km^2^ in JJA 2002 before recovering the following summer to +0.62 × 10^6^ km^2^. In DJF and MAM 2006, sea ice extent shrank to values of −0.67 × 10^6^ km^2^ and −0.82 × 10^6^ km^2^, respectively, before recovering to a positive value in JJA 2006 of +0.11 × 10^6^ km^2^ and an SON value of +0.52 × 10^6^ km^2^. In summer and fall of 2011, sea ice extent values had dropped to −0.07 × 10^6^ km^2^ and −0.53 × 10^6^ km^2^, respectively, before recovering to +0.38 × 10^6^ km^2^ in SON 2011. Therefore, the sustained lack of recovery in the recent 2-year period is remarkable, and suggests that there must have been significant changes in the upper ocean that played a major role in the rapid reduction of sea ice extent.

### Role of the ocean

**Fig. 3:**
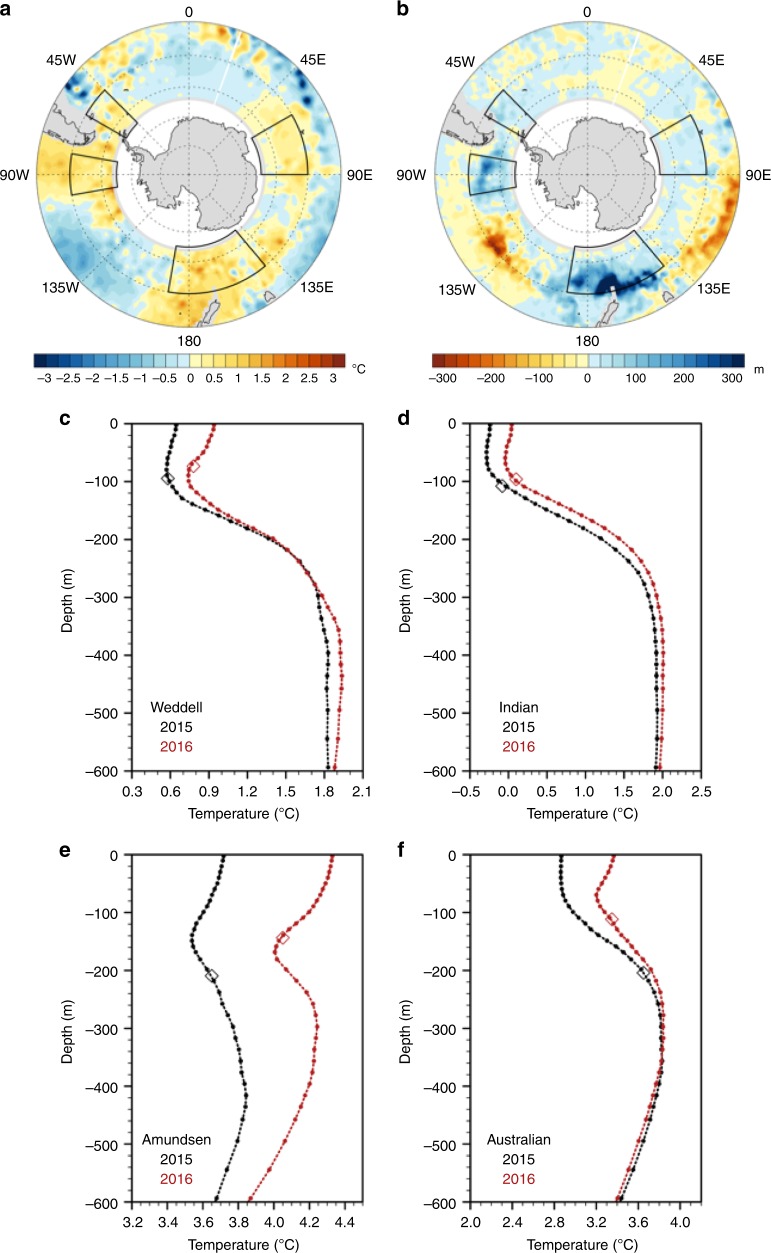
Characteristics of the Southern Ocean in 2016 compared to 2015. **a** Gridded Argo float 2.5 m temperature differences (°C), SON 2016 minus SON 2015^33^; **b** same as (**a**) except for mixed-layer depth anomalies (m); SON 2015 (black) and SON 2016 (red) vertical temperature profiles (°C) in the upper 600 m and mixed-layer depth (diamonds) from the gridded Argo float data averaged over the (**c**) Weddell region (50–65°S, 45–60°W), **d** Indian region (50–65°S, 60–90°E); **e** Amundsen region (50–65°S, 80–100°W); and **f** Australian region (50–65°S, 140°E–170°W). The regions are denoted in (**a**) and (**b**)

As noted above, the changes in both the sea ice extent and in SSTs at mid- and high southern latitudes persisted beyond SON 2016 (Fig. 1b, c). The 1-year change of temperatures at 2.5-m depth for SON 2016 minus SON 2015 from gridded Argo float data shows local warming of nearly 2 °C in the northwest Weddell Sea, Amundsen Sea, south of New Zealand and Australia, and in the southern Indian Ocean (Fig. 3a).

The regions outlined in Fig. 3a correspond to areas of anomalous northerly winds and areas of greatest sea ice retreat (Fig. 2a). In those same regions, there are shallower mixed-layer depths in SON 2016 compared to SON 2015 (Fig. 3b). The warmer upper ocean and shallower mixed layers for those four regions are shown in temperature profiles of the upper 600 m of the ocean (Fig. 3c, d, e, f). The fact that these changes are present not only at the surface but also extend to such depths suggests that the conditions in SON 2016 could have been the culmination of processes occurring on longer timescales. That is, the warmer water at depth could have been moved upward nearer the surface where a triggering event with low SAM/positive IPO could have produced the SST transition. The climatological mixed-layer depths based on an Argo climatology^20^ reach maximum depths of 156, 214, 156, and 133 m averaged over the Australia, Amundsen, Weddell, and Indian regions, respectively (Supplementary Figure 7). For all regions, the maximum mixed-layer depth occurs during August. Warm water, which is brought upward by changes in Ekman suction, is likely to be entrained into the surface layer in late winter when the mixed layer is deepest contributing to warming of that layer. Thus, the trend in wind stress curl on decadal timescales associated with negative IPO/positive SAM produces upward advection of warmer subsurface water through Ekman suction. Episodic entrainment of that warmer water below the mixed layer into the surface layer during late winter would then precondition the rest of the season to be warmer, and over time would produce a relatively warmer water column in the upper ocean.

Anomalous westerlies for positive SAM and negative IPO (conditions seen, on average, in the early 2000s) would both promote expanding sea ice that corresponded to the accelerated rate of positive sea ice extent in that time period^1,21^ (Fig. 1a). These conditions also drive Ekman transport of cooler surface water equatorward and promote upward Ekman suction around Antarctica. As noted above, this upwelling (Ekman suction) brings warmer subsurface water toward the surface. Then, when easterly anomalies occur with negative SAM in SON 2016, wind stress curl is positive, warmer surface water is transported south, SSTs increase, and the entire water column is then warmer as evidence of the culmination of these processes. The background decadal timescale upward movement of warmer water to near the surface is related to the two timescale response previously mentioned in association with the SAM^22–24^ when negative IPO and positive SAM are acting in the same direction. Then there is the interannual negative SAM conditions in SON 2016 that contribute to warming the SSTs.

**Fig. 4:**
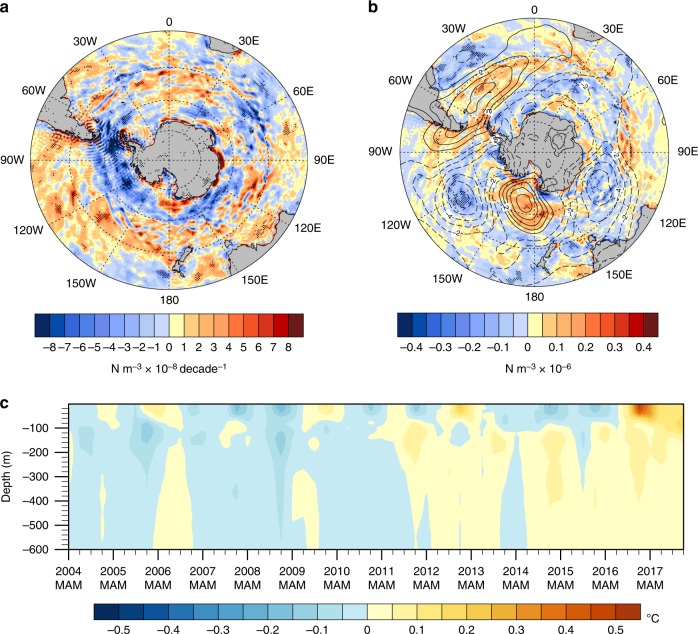
Changes in wind stress curl and Southern Ocean response. **a** Linear trend from 2000 to 2014 of wind stress curl (N m^−3^ ×  10^−8^ decade^−1^), computed from ERA-Interim wind stress, negative trend values denote areas of Ekman suction that would move water upward in the column; **b** SLP anomalies for SON 2016 (hPa, as in Fig. 2a) with SON wind stress curl anomalies superimposed as colors (N m^−3^ × 10^−6^); **c** zonal mean seasonal temperature anomalies (°C) relative to 2004–2017 climatology from gridded Argo float data for upper 600 m averaged over 50–65°S

To explore this idea further, the trend of wind stress curl is negative in most regions between 50 and 65°S over the time period when the IPO was in its negative phase and the SAM was trending positive (Fig. 4a). Wind stress curl trends greater than roughly 3 × 10^−8^ N m^−3^ decade^−1^ are significant at the 10% level. The negative trend in wind stress curl would produce a trend in Ekman suction resulting in more upwelling of warm subsurface water over time (see Methods).

For values of the trend in wind stress curl near 60°S from Fig. 4a of about 1 × 10^−7^ N m^−3^ yr^−1^, the average change of vertical velocity from Ekman suction (*w*_e_ in Methods) over the last decade was about 0.5 × 10^−6^ m sec^−1^. This would amount to an addition of about 15 m per year of upward vertical motion driven by the wind, or 150 m if applied over 10 years. For the largest values of wind stress curl trend in Fig. 4a of around 7 × 10^−7^ N m^−3^ yr^−1^, this would give about 100 m per year of upward vertical motion in some locations. This estimate does not take into account changes in the diapycnal/eddy-driven upwelling that would also respond to changes in the wind stress.

**Fig. 5:**
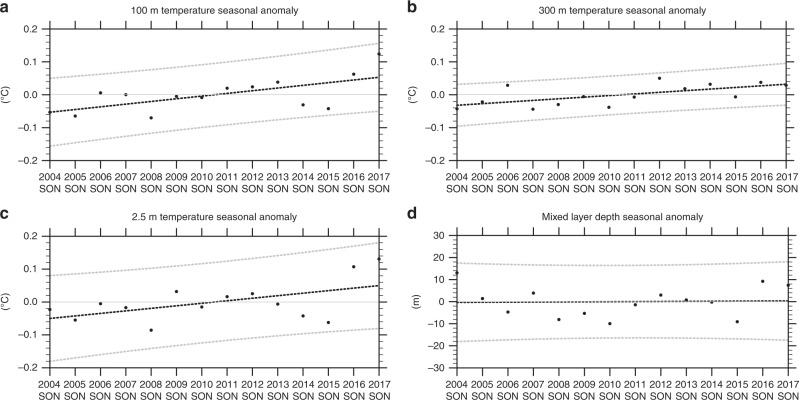
Decadal timescale trends in the Southern Ocean. Time series of SON seasonal anomalies relative to 2004–2017 climatology averaged over 50–65°S from gridded Argo float data for **a** temperature (°C) at 100-m depth showing a linear trend of +0.08 °C decade^−1^, *r*^2^ value of 0.41, significant at the 1% level; **b** temperature (°C) at 300-m depth showing a linear trend of +0.05 °C decade^−1^, *r*^2^ value of 0.40, significant at the 1% level; **c** temperature (°C) at 2.5-m depth showing a linear trend of +0.08 °C decade^−1^, *r*^2^ value of 0.28, significant at the 5% level, with the low *r*^2^ value associated with the step increase of temperature in SON 2016; **d** mixed-layer depth (m) showing a linear trend of 0.5 m decade^−1^ with an *r*^2^ value near zero

For zonal mean seasonal temperatures averaged from 50 to 65°S from the gridded Argo float data, there is evidence of a decadal-timescale movement of warm water upward with interannual variability superimposed (Fig. 4c). SON 2016 shows the first instance in the record of positive anomalies throughout the column down to 600 m. These are then sustained through the subsequent year (2017). To quantify the decadal-timescale warming trend below the surface, the time series of SON temperature anomalies from Fig. 4c at 100-m depth shows a linear trend +0.08 °C decade^−1^ and an *r*^2^ value of 0.41 significant at the 1% level (Fig. 5a; see Methods). The time series for 300-m depth shows a linear trend of +0.05 °C decade^−1^ and an *r*^2^ value of 0.40 significant at the 1% level (Fig. 5b). Meanwhile, there is little warming trend in the 2.5-m depth layer until temperatures jump from a value in SON 2015 of nearly −0.1 °C, to +0.1 °C in SON 2016, the largest interannual SON increase in the record (Fig. 5c). Since the largest contributions to the long-term trend occur in the last 2 years, this step increase produces an *r*^2^ value of 0.28, about half the *r*^2^ values for 100 and 300 m. Mixed-layer depths (Fig. 5d; see Methods) also do not show much trend prior to SON 2016 when the anomaly value of about +10 m occurs.

Removing the 2017 endpoints from these time series produces warming trends that are significant at the 7% level for 100 m, and 3% at 300 m. The trend at the surface is not significant with the endpoint removed, which is indicative of the upward warming jump near the end of the time series. The similar interannual variations of 100 m and 2.5-m temperatures (Fig. 5a, c) are evidence of the episodic warming of the surface layer superimposed on the positive decadal trend. The strong teleconnection pattern in SON 2016, associated with negative SAM and positive IPO, produces positive wind stress curl values of around +0.5 N m^−3^ × 10^−6^ (and associated southward Ekman transport at the surface) in areas of the southeast Pacific, near New Zealand, south of Australia, and south of India (note the areas highlighted in Fig. 3 with correspondence to those areas in Fig. 4b), and a rapid increase in SSTs and 2.5-m temperature.

Though there could be other interpretations, a plausible one supported by evidence presented here indicates that the Southern Ocean was conditioned by a 15-year trend of negative wind stress curl that, through associated Ekman suction, was bringing warmer water higher in the water column. The base state differences between the period of negative IPO from 2000 to 2014 minus the previous positive IPO period show a buildup of warm water anomalies of nearly +0.5 °C below about 200 m, and cold water anomalies approaching −0.3 °C above 200 m south of about 50°S (Fig. 1b). Then in SON 2016, the strong teleconnection pattern, driven by the record precipitation near 120°E, Eq, was sufficient to force surface wind anomalies that produced regional positive wind stress curl anomalies and anomalously warm water at the surface. Combined with the warmer water that had made its way to the upper part of the water column in regions south of 50°S, there was then a warmer upper ocean column from the surface down to 600-m depth that contributed to the sustained retreat of Antarctic sea ice (Figs. 1c, 4). The upper ocean at high southern latitudes remained warm in 2017, and sea ice extent remained low (Figs. 1, 4). This hypothesis is complementary to the proposed relationship between expanding (declining) sea ice and ocean warming (cooling) at depth, suggesting that the expanding sea ice itself may have led to a buildup of warm water at depth that could be quickly released to the surface should a reversal in sea ice occur^25^.

## Discussion

The evidence indicates that the rapid decrease in Antarctic sea ice extent in late 2016 and the significant changes in the upper Southern Ocean were likely caused by three factors. First, record SST, precipitation, and convective heating anomalies in the eastern tropical Indian and far-western Pacific Oceans produced an anomalous Rossby wave response in the mid- and high southern latitudes in SON 2016. The consequent teleconnection pattern around Antarctica was characterized by a record negative phase of the SAM, and a preponderance of warm, moist southward surface winds that drove sea ice southwards and produced decreased sea ice extent. Second, the anomalous surface winds associated with this teleconnection pattern were also associated with positive wind stress curl anomalies, southward Ekman transport, and warmer surface water transported southward. Third, a decadal timescale trend of negative wind stress curl anomalies over the 2000s, associated with the positive trend of the SAM and the negative phase of the IPO, moved warmer subsurface water in the Southern Ocean upward in the column (part of the so-called two-timescale response^22–24^). Then in late 2016, the negative SAM contributed to producing anomalously warm SSTs such that the entire upper 600 m (over many areas of the Southern Ocean) then was characterized by positive temperature anomalies. These warmer ocean temperatures, combined with the direct effects of surface wind forcing on the sea ice, produced the rapid decrease of Antarctic sea ice extent. These conditions were maintained through 2017, with reduced sea ice extent compared to the average of the 2000s through JJA 2018.

## Methods

### IPO calculation

The IPO is calculated as the second EOF of observed SSTs^26^ from 1900 to 2014 that are first low-pass filtered using a Lanczos filter with a 13-year low-pass cutoff (using seasonal data with 73 weights): https://www.ncl.ucar.edu/Document/Functions/Built-in/filwgts_lanczos.shtml ^7^.

### Trend calculation

The linear trends and *t*-test calculations in Fig. 5 are described in https://www.ncl.ucar.edu/Document/Functions/Built-in/regCoef-1.shtml.

### SAM index

The SAM index is defined as the zonal mean SLP difference, 40°S minus 60°S^27^.

### Ekman suction calculation

A rough estimate of the magnitude of Ekman suction, *w*_e_, can be obtained by

$${w}_{\mathrm{e}} = \frac{1}{{\rho f}}\nabla \times \tau \cdot \hat k$$

where the density of seawater *ρ* and Coriolis parameter *f* = 2*Ω* sin*θ* where *Ω* is the rotation rate of the planet and *θ* is the latitude where *f* is negative in the Southern Hemisphere. The density of seawater *ρ* is about 1025 kg m^−3^, while *f* in mid-latitudes is about 1 × 10^−4^ sec^−1^, and $\nabla \times \tau \cdot \hat k$ is the vertical component of the curl of the wind stress. In the long-term mean, upwelling driven by the wind is balanced by diapycnal (mixing) and eddy-driven overturning allowing the density structure to be in the steady state. However, with a trend in *w*_e_ that is not balanced by changes in the eddy/diapycnal-driven overturning, the pycnocline would be expected to move in the vertical.

### Specified convective heating anomaly experiment

The specified convective heating anomaly experiment with the Community Atmospheric Model version 3 (CAM3) is run here at T42 resolution with climatological monthly varying SSTs^1^. We define a local heat source in the same position as the prominent precipitation anomalies that would produce such convective heating anomalies. A positive convective heating anomaly (corresponding to a positive precipitation anomaly) is placed in the CAM3 in the shape of an elongated ellipse in the eastern Indian Ocean region centered on the Equator, 120°E, where increased precipitation occurs (Fig. 2d). This heating has a central value of +5°C day^−1^ and decreases linearly with distance until it vanishes on an elliptical boundary with a 3000-km east–west semimajor axis and a 500-km north–south semiminor axis. In the vertical, the heating has the profile sin(π*p*/ps), where *p* is pressure and ps is surface pressure. This profile is an approximation to the vertical distribution of long-term heating anomalies that tend to occur in this model. At the center, the magnitude of the idealized source produces the same depth-averaged heating as about a 5 mm day^−1^ precipitation anomaly. This is comparable to the magnitude of the largest anomalies in the GPCP observations at that location (Fig. 2d). Thus, the midlatitude response should be representative of that in the observed system. The experiment was run for 30 years, and the results are shown as anomalies from a 100-year control run that uses specified climatologically varying sea surface temperatures. The larger value of the SAM simulated in the model experiment for SON (−1.12) compared to the observed value in SON 2016 of −0.52 suggests that if just forcing from the tropical eastern Indian Ocean was acting in the observed system, the SAM could have been even more anomalously negative. But in the real system, there are influences from other regions that are affecting the high-latitude circulation that could be inferred to make the SAM less negative during this period.

### Mixed-layer depth calculation

We use a sigma criterion of 0.03 kg m^−3^ difference in density at depth from the surface to calculate mixed-layer depth in the Argo data^28^. This method is widely used^28,29^ and is consistent with recent work using Argo^30^.

### Code availability

The climate model code and the scripts used to generate the plots in this paper are available from the corresponding author on request.

## Supplementary information

Supplementary Information

## Data Availability

Sea-ice extent data in Fig. 1 are pre-calculated monthly extent values from the.csv files in Version 3 from ftp://sidads.colorado.edu/DATASETS/NOAA/G02135/ that are qualitatively comparable to the quality-controlled files that end in February 2017. EN4 reanalysis data are available from https://www.metoffice.gov.uk/hadobs/en4/download-en4-2-0.html#l09_analyses, and ERA-Interim wind stress from http://apps.ecmwf.int/datasets/. The sea-ice concentration data from NASA are available at ftp://sidads.colorado.edu/pub/DATASETS/nsidc0051_gsfc_nasateam_seaice/final-gsfc, and the Argo float data are at http://sio-argo.ucsd.edu/RG_Climatology.html. The SAM index is from http://www.nerc-bas.ac.uk/icd/gjma/sam.html. GPCP precipitation data are available from http://www.esrl.noaa.gov/psd/data/gridded/data.gpcp.html. Hurrell et al. SST data can be found at https://climatedataguide.ucar.edu/climate-data/merged-hadley-noaaoi-sea-surface-temperature-sea-ice-concentration-hurrell-et-al-2008. The specified convective heating anomaly experiment model data are on the NCAR mass storage system and can be made available from the corresponding author on request.
