# Supplementary material for: Sustained ocean changes contributed to sudden Antarctic sea ice retreat in late 2016
Source: Nat Commun. 2019 Jan 2;10:14. doi: 10.1038/s41467-018-07865-9 (PMC6312884; doi:10.1038/s41467-018-07865-9)
Supplement: Supplementary file 1 — Supplementary Information [file 41467_2018_7865_MOESM1_ESM.pdf]

Supplementary Information

Recent sudden Antarctic sea ice retreat caused by connections to the tropics and sustained ocean changes around Antarctica

Meehl et al.

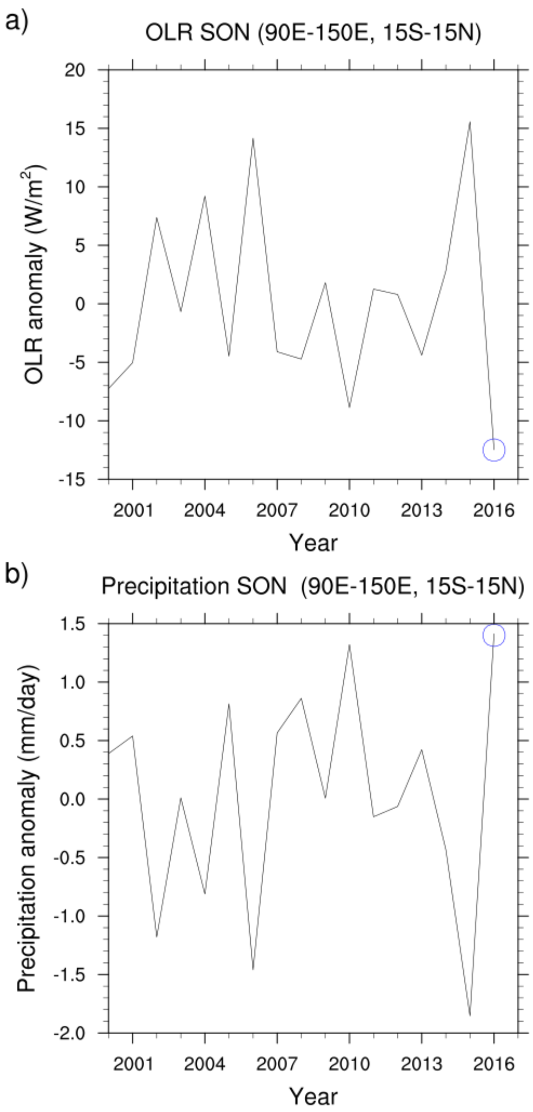

Supplementary Figure 1: a) outgoing longwave radiation anomalies (OLR, W m^{-2}) for SON, 2000-2016, relative to the 2000-2014 base period from the NCAR/NCEP reanalysis (https://www.esrl.noaa.gov/psd/data/gridded/data.ncep.reanalysis.html), for the region 90°E-150°E, 15°S-15°N; value for SON 2016 circled; b) same as (a) except for precipitation plotted from 2000-2017 (mm day^{-1}), SON 2016 circled.

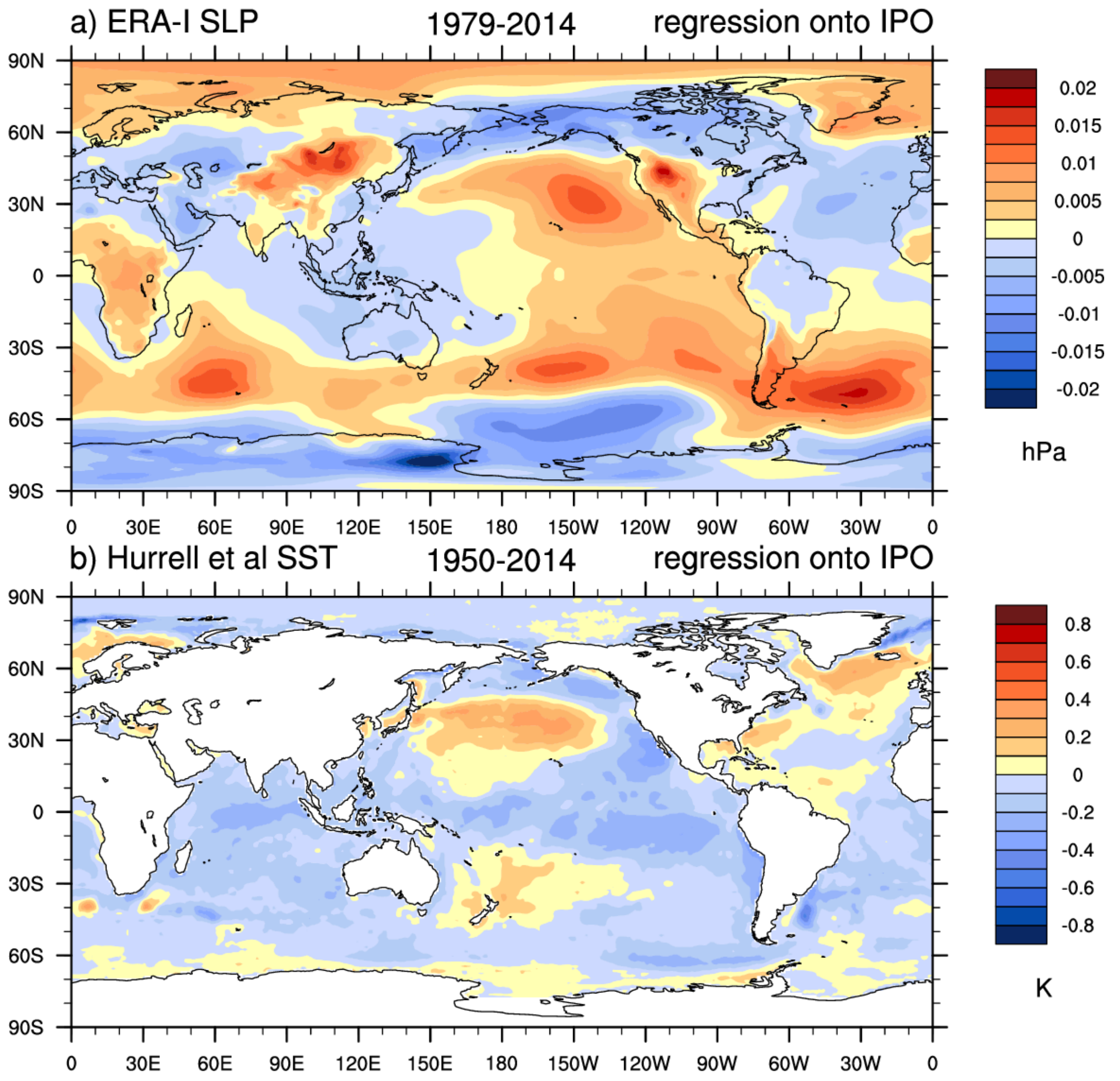

Supplementary Figure 2: a) Regression of SLP onto the IPO time series (1979-2014, representing the most recent positive and negative IPO periods, after ref 1), the sign convention for SLP anomalies is for negative IPO; b) regression of SSTs onto the IPO index for the time period 1950-2014 when data are somewhat more reliable over the Southern Ocean; the sign convention of the SST anomalies is for negative IPO.

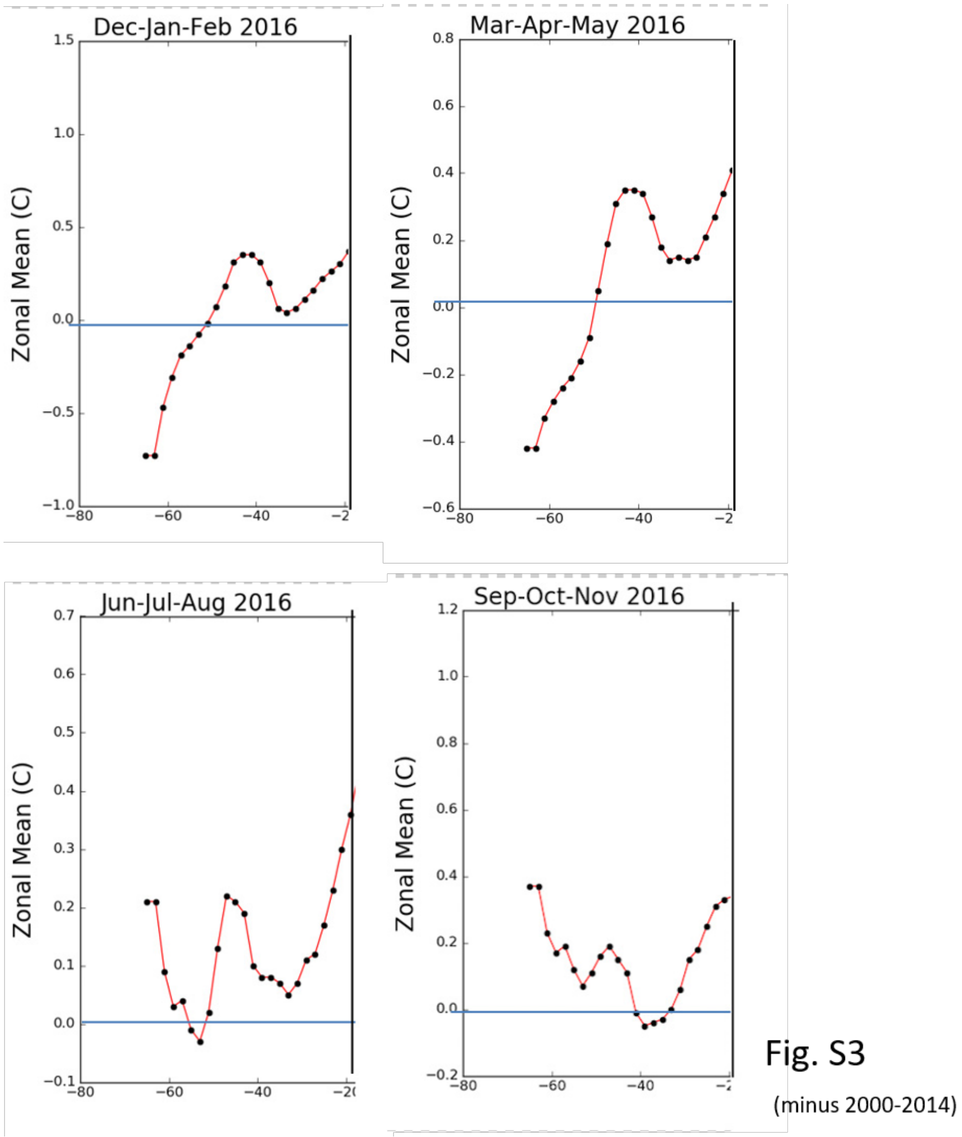

Supplementary Figure 3: Seasonal zonal mean SST anomalies showing the rapid transition of negative zonal mean SST anomalies (˚C) south of about 50˚S in the first half of 2016, to positive zonal mean SST anomalies south of 50˚S later in 2016; a) DJF 2016 minus 2000-2014 base period; b) same as (a) except for MAM 2016; c) same as (a) except for JJA 2016; d) same as (a) except for SON 2016 (SSTs from ref 26).

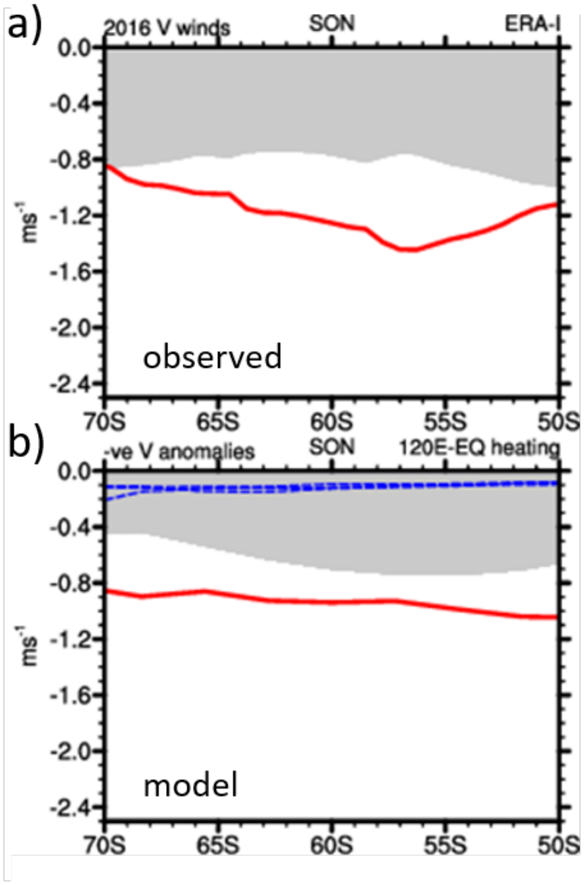

Fig. S4

Supplementary Figure 4: a) Following reference 1, except for zonally averaged negative v-component surface wind anomalies (m sec^{-1}), red line is SON 2016, shading is measure of noise; b) same as (a) except red line for model experiment with positive convective heating anomaly centered at 120˚E, Eq., blue dashed lines are 30 year periods in control run.

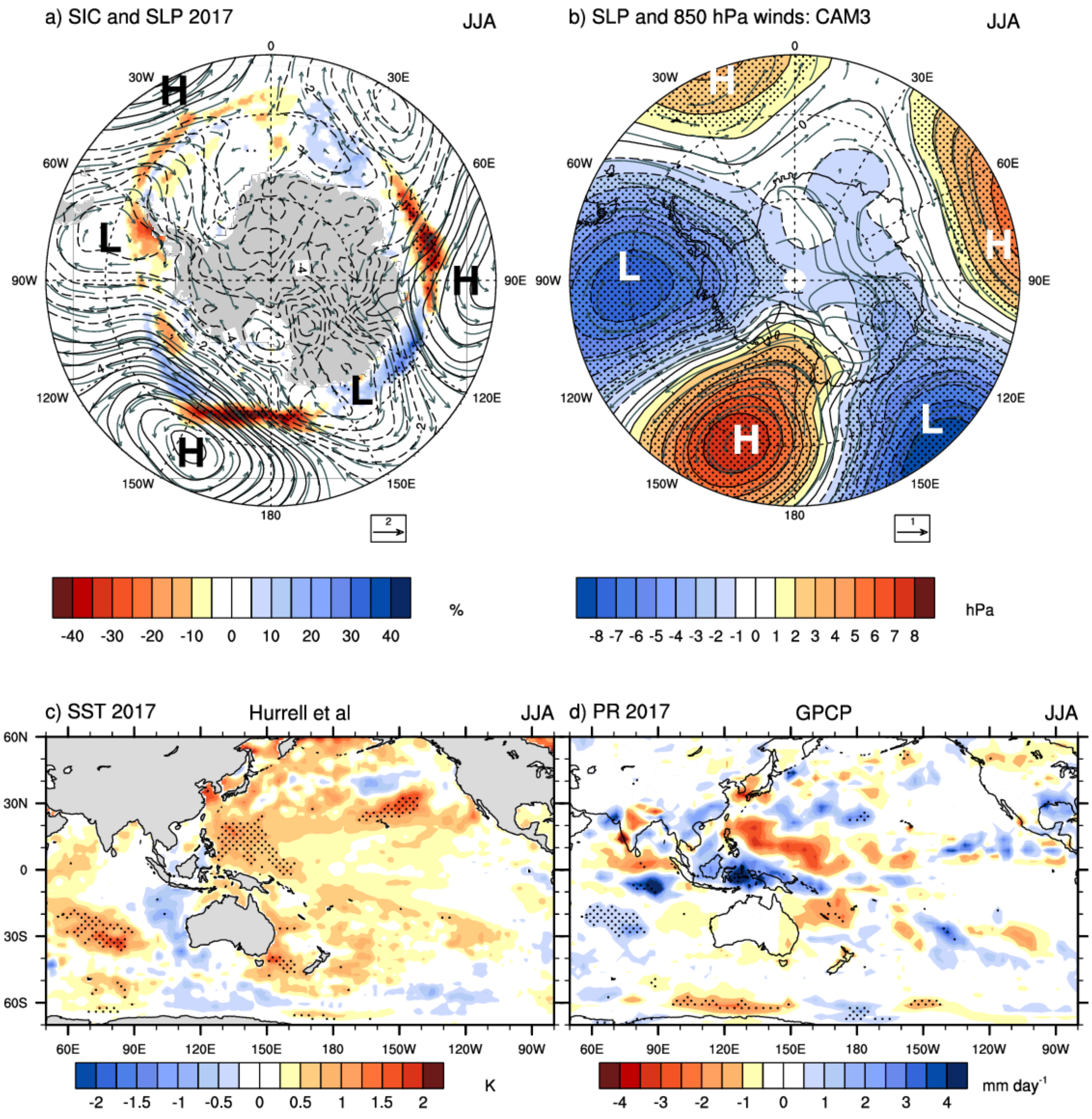

Supplementary Figure 5: Same as Fig. 2 in main text except for June-July-August 2017. Note observed SAM index for JJA 2017 is +0.82, and SAM index from model simulation is +0.87.

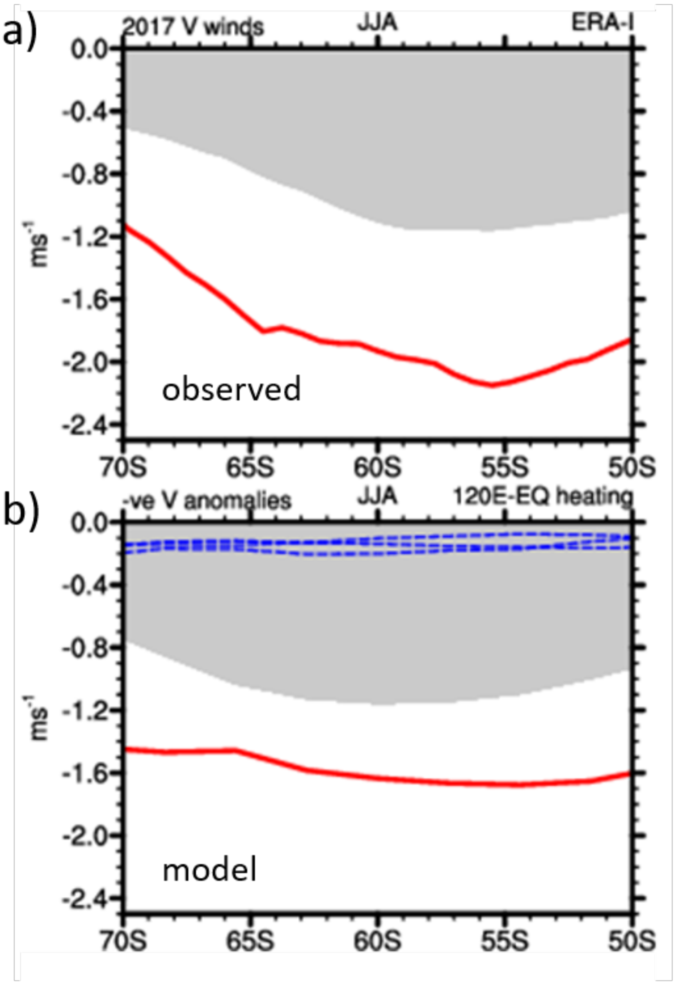

Fig. S6

Supplementary Figure 6: a) Following reference 1, and same as Supplementary Figure 4 except for red line is JJA 2016 for zonally averaged negative v-component surface wind anomalies (m sec^{-1}), shading is measure of noise; b) same as (a) except red line for model experiment with positive convective heating anomaly centered at 120˚E, Eq., blue dashed lines are 30 year periods in control run.

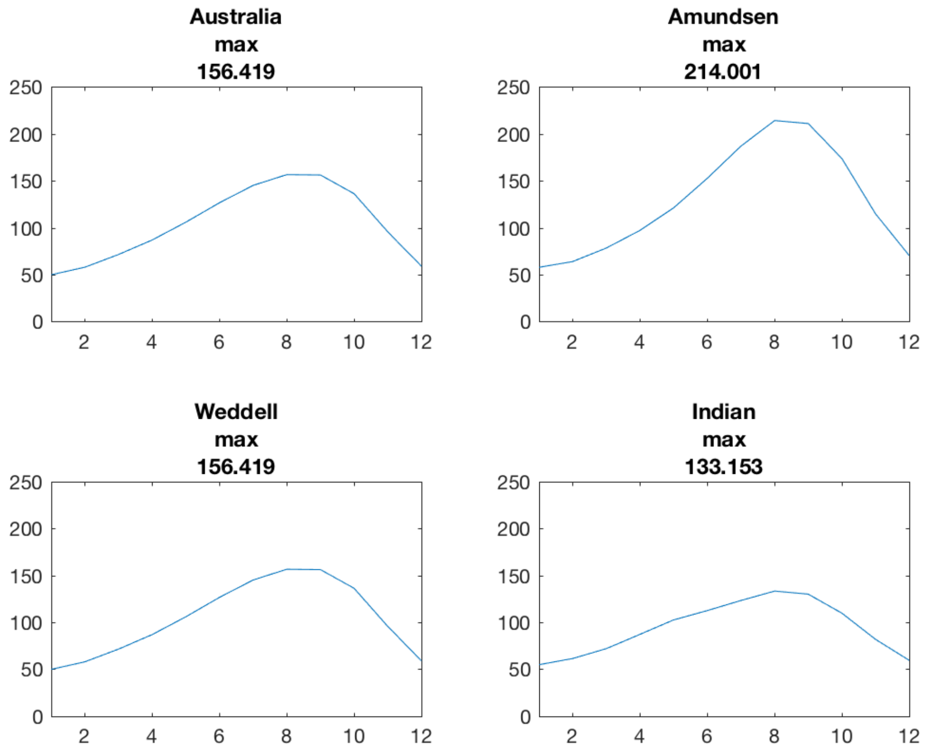

Supplementary Figure 7: The climatological mixed-layer depth based on an monthly mean Argo climatology (20) averaged over the Australia, Amundsen, Weddell, and Indian regions, respectively, as outlined in Fig. 3a,b. Maximum monthly mean values for each area noted in panel label and all occur in August; x axes are months; y axes are depth (m).
